# In this month’s Bulletin

**DOI:** 10.2471/BLT.20.000620

**Published:** 2020-06-01

In the editorial section, Philip Setel et al. (374) emphasize the need for rapid mortality surveillance during a pandemic. Jack Collins et al. (375) review ethical considerations of using mystery shoppers to study pharmaceutical sales.

In the news section, Lynn Eaton and Gary Humphreys (378–379) report on calls to address the limitations of the *International Health Regulations*. João Aprígio Guerra de Almeida speaks to Andréia Azevedo Soares (380–381) about the origins of Brazil’s human milk bank network and the psychosocial aspects of breastfeeding.

## African region

### Reproductive, maternal, newborn and child health interventions

Fernando C Wehrmeister, et al. (394–405) estimate inequalities in coverage.

### Making organ transplants possible

André Loua et al. (420–425) compare donation policies and programmes.

## Brazil, Ghana, Mexico 

### Raising healthy babies 

Mireya Vilar-Compte et al. (382–393) estimate costs of maternity leave to support breastfeeding.

## China

### Reducing the prevalence of myopia

Catherine Jan et al. (435–437) focus on prevention efforts.

## Latvia

### Providing mental health care

Maris Taubea and Wilm Quentin (426–430) study the impact of community-based programmes.

## Global

### Antibiotics for children 

Shrey Mathur et al. (406–412) compare five dosing guidelines.

### WHO’s prequalification programme

Ariadna Nebot Giralt et al. (413–419) survey nongovernmental organizations. 

### Preventing sexually transmitted infections

Igor Toskin et al. (431–434) propose combining behavioural and biomedical interventions.

### Measuring premature deaths

Shah Ebrahim et al. (438–440) argue for improvements to the indicator definition.

